# Hand-Book for the Military Surgeon

**Published:** 1861-09

**Authors:** 


Art. V.—*Hand-Book for the Military Surgeon: being a Compendium of the Duties of the Medical Officer in the Field, the Sanitary Management of the Camp, the Preparation of Food, etc.; with Forms for the Requisitions for Supplies, Rations, etc.; the Diagnosis and Treatment of Camp Dysentery; and all the important points in War Surgery: including Gunshot Wounds, Amputation, Wounds of the Chest, Abdomen, Arteries, and Head, and the Use of Chloroform.* By Charles S. Tripler, A.M., M.D., Surgeon U.S.A., and George C. Blackman, M.D., F.R.M.S., etc., etc. 12mo., pp. 163. Cincinnati: Robert Clarke & Co., 1861.

*A Practical Treatise on Military Surgery.* By Frank Hastings Hamilton, M.D., etc., etc., etc. 8vo., pp. 232. New York: Bailliere Brothers, 1861.

Very few, even of those best qualified for the practice of civil surgery, would be found capable of entering at once upon the army medical service without some hints derived from the experience of those who have already labored in that field; it is, therefore, in the due course of things, that, at the present juncture of our national affairs, when a large volunteer force is to be raised, and must be accompanied by competent surgeons, attempts should be made to assist these gentlemen in preparing for their new duties; and even then there must be many things which every man must learn for himself, in the actual discharge of his functions.

The first of the books named in the heading of this notice is, as it purports to be, a hand-book, and a very good one. Its scope is, to a great extent, set forth upon its title-page, and we are sure that it will prove of essential service to those for whose use it is intended. There is not within our knowledge a more comprehensive or convenient manual on the subject. Dr. Hamilton's work, while it contains a great deal of valuable information, bears evidence of the haste with which it was prepared, and does not really come up to its author's usual standard of accuracy and thoroughness, at least in that portion devoted more exclusively to professional matters. It is somewhat copiously illustrated, and in this respect has the advantage of its fellow in the present notice. We can honestly recommend those who are about entering upon the medical service of the army to study carefully both these works, perhaps in preference to the more extended treatises of Hennen, Ballingall, Guthrie, Macleod, and others. Or rather, let these essays be used as guides to the general comprehension of the subject, so that the novice may be at least saved from disgracing himself; the experience of such men as those just named may and should be afterward drawn upon as much as possible, when time permits, and their value can be appreciated. It only remains for us to comment briefly upon a few practical points, which have occurred to us in connection with the various topics discussed by our authors.

The surgery of the army differs in some respects from that of civil life, although its fundamental principles are precisely the same. An army surgeon sustains a continual responsibility for the hygienic regulations of the troops under his charge, and must understand just how far, and by what means, it is his duty to modify or control them; in this respect he has much more his own way than a civilian can ever have. He has usually a special set of diseases and injuries to deal with, and under circumstances which, always peculiar, may become in the highest degree trying to his skill and judgment. After an engagement, a greater or less number of wounded men are thrown upon his hands; and to discharge his duty toward them properly, may demand the utmost exercise of his energy and discretion. No amount of fatigue, hunger, thirst, discomfort, or danger must ever deter a surgeon in any sphere from fulfilling his functions; but these hinderances are far more commonly met with in military than in civil practice.

In both the volumes before us we find much that is valuable in regard to the hygienic management of troops. Dr. Hamilton has gone a step behind this, giving a chapter on the examination of recruits, in preparing which he acknowledges his indebtedness in great measure to the "Manual" of Dr. Tripler, adopted by the War Department. Dr. H. has also entered somewhat at length into the subject of the diet, clothing, and lodging of the soldier, in and out of hospital; he gives some useful illustrations in connection with his remarks on the latter of these topics.

It can hardly be necessary to insist upon the importance of such matters, which might indeed be usefully discussed in a separate volume. Too often the surgeon, either from indolence, from an aversion to interfering with what might seem to be without the sphere of his duty, or from a consciousness of a want of practical knowledge, withholds the ounce of prevention, and in due time is called upon to administer the pound of cure. Then, perhaps, having it imperatively forced upon his notice that an infringement of hygienic laws, with its unfailing retribution, constitutes the rationale of the claim upon his services, he learns to avoid any repetition of the evil. Undoubtedly, there are cases in which the risk to health must be run, just as the risk of being shot in battle must be, or where the unfavorable condition is beyond reach; but we feel certain that an earnest and continued vigilance on the part of the surgical staff of the army would go far to lessen the suffering and mortality incident to the carrying on of any war.

One fact must, however, be considered with regard to the diet and clothing of soldiery; these matters are under the charge of officers of the quartermaster's and subsistence departments, and are provided for in the Army Regulations, in general terms. Hence, as for instance in regard to uniforms, it may be competent for the surgeon to suggest, but not to do more. It would, perhaps, be well if the medical officers were allowed greater influence in the arrangement of these things, although there is no doubt that this depends in the case of each surgeon upon his established character for good sense or otherwise, and upon his tact and judgment in dealing with other functionaries. The genius of some nations leads them to adhere obstinately to what they are accustomed to, even if its disadvantages are manifest; but such is not the tendency of our countrymen, and we hope to see all the teachings of experience made useful for promoting the health, comfort, and efficiency of our soldiers.

Let us note in passing, that Dr. Hamilton says "broad flannel bands, placed around the abdomen, make tolerable substitutes for shirts." This is very true; but we would extend the application of these bands, and have them used by every soldier, with or without a shirt, if his duty calls him to serve in hot or variable climates or seasons. Sure are we that this is one of the most certain and effective precautions against those disorders of the abdominal viscera which have disabled or destroyed so many men under such circumstances.

Dr. Hamilton has done very good service, we doubt not, to many a young army surgeon, in writing as he has upon the various methods of lodging soldiers. It is singular how many sensible men are entirely ignorant of the practical details of this subject. For instance, in regard to the sinks for a camp, although their position is prescribed in the Army Regulations, the mode of arranging them is left undecided. Dr. Hamilton says "they should be narrow and deep, so as to leave as little surface for evaporation as possible; and every day a certain amount of the earth excavated in their construction should be replaced until the whole is covered in, and then new sinks should be dug. When latrines can be placed over running water this necessity is obviated." Dr. Tripler says "the usual method of constructing these sinks is to dig a shallow trench, some eight or ten feet long, to drive a strong forked stake at each end, and to place a strong pole, from one fork to the other, for the men to sit on. These sinks should be at least five feet in depth; immediately after reveille, not less than six inches of earth should be thrown upon the deposits of the preceding day and night, and when they are filled to within two and a half feet of the surface, they should be completely filled up and new sinks dug." Simple and natural as this plan is, it does not necessarily suggest itself to the mind; we have several times asked intelligent civil practitioners how they suppose the matter is managed, and found them unaware even that any special provision was made for it. On this and analogous points, it is of importance that the surgeon should be informed beforehand; for, although there will almost certainly be found in any body of troops men who know from experience how all these things are done, yet these very men will be apt to neglect their duty, or to perform it improperly, unless they have competent overseers.

One omission, which so far as we know, may be charged to every work hitherto published on military surgery, is really surprising. No mention is made, by any of these writers, of "sunstroke," as it is called, to which troops on the march or on parade are so constantly liable in hot weather, and by which so many lives have been lost.

We cannot but be struck with the slight stress laid by either of our authors upon external applications in the treatment of sick or wounded men. Even in speaking of dysentery, in which dry heat, frictions, fomentations, poultices, sinapisms, or blisters, applied to the abdomen, are so widely used in civil practice, Dr. Hamilton mentions none but internal remedies; and the only outward medication alluded to by Dr. Tripler is that by cupping. Stimulants given by the mouth are relied upon altogether by Dr. T. in the treatment of collapse; but no civil surgeon who sees much of accidents by railroad or machinery—the class of injuries most nearly allied to gunshot wounds—would willingly abandon the use of cutaneous irritation in the shock by which those accidents are so invariably followed. Dr. Hamilton does not speak at all of treating this condition.

Upon the subject of amputation, our authors are agreed in advising that the operation should be primary. Hamilton would restrict the employment of the flap method to cases in which every moment of time is of importance; Tripler recommends its adoption in the arm and thigh, but is in favor of the circular for the forearm and leg. We would suggest that perhaps the operation depends for its success far more upon the after-treatment, and upon the attention paid to the moving, etc., of the patient, than to what we cannot but consider as trifling modifications of the process of amputation. In making this remark, we have reference to military practice alone. Here existing circumstances are apt to narrow our aims down to the saving of life; and desirable as it may be that every stump should prove suitable for the fitting of an artificial limb, this is a point of less moment than in civil surgery. In judging of the success of army practice, we consider simply the lives saved and lost; in civil practice, we add to the calculation some niceties which must be foregone on the battle-field. A sufficiency of soft parts to more than cover the bone, the thorough ligation of every bleeding vessel, and such a plan of dressing as will completely protect and support the stump,—these are the great points to be aimed at; if they are gained, and the patient safely conducted through the grim array of secondary symptoms, hemorrhage, gangrene, erysipelas, purulent infection, tetanus, the amputation is a successful one. In our own opinion, the less amount of disturbance of the natural relations of the parts in the flap operation gives it a decided advantage over the circular, as usually performed.

As regards anæsthesia, Hamilton prefers ether to chloroform, but thinks both forbidden "when the system is greatly prostrated by disease, or by the shock of a recent injury, unless the patient exhibits an unconquerable dread of the pain of the operation, or the operation is likely to prove exceedingly painful. It is our opinion also," says he, "that anæsthetics sometimes, and especially chloroform, prevent the union of wounds by adhesion, or by 'first intention.'" Dr. H., however, elsewhere attributes the want of success of the Crimean surgeons in obtaining the early healing of their stumps, to their employment of the flap method in amputating, rather than the circular. Tripler and Blackman simply transcribe the remarks of Mr. Macleod, in his "Notes on the Surgery of the Crimean War," in favor of the use of chloroform. So far as our own experience goes, in both hospital and private practice, a combination of ether and chloroform, three parts to one, is an anæsthetic at once safe and efficient.

The subject of resection or excision, as a substitute for amputation, is one which might, we think, have been properly discussed at much greater length than it has been in these works; although Dr. Tripler would seem to limit its applicability to the joints of the upper extremity, particularly the elbow.

Many other topics suggest themselves to us for mention, but we must forbear at present, on account of the limited space we have at command. We would simply hint that, in the fresh editions of these volumes, which are so likely to be soon called for, the authors would do well to insert some general directions as to the order in which a number of wounded men, as after an engagement, should be attended to; so that the novice may not deliberately amputate the little finger of the first man in a row, while the second bleeds to death from the femoral artery, because he was the second, and must wait his turn. It would be well also for them to give directions for one or two of the most generally accepted methods of performing each operation, so that the surgeon may never be without a guide, should his memory fail him at a pinch. The adoption of a special type for these directions, or the grouping of them together at the end of the volume, would make it easier to turn to them at once.

Our readers are probably aware that measures have been taken by the proper authorities to insure the exclusion of unqualified persons from the medical service of our volunteer forces. This judicious movement may be said to be met, on the other hand, by those who have issued, at the present juncture, aids to those who are about to undertake the duties of that service. Of all the various paths open to the willing servants of their country, that trodden by the military surgeon is one of the most arduous and responsible, and one of the least likely to lead to earthly glory; but he is none the less sure of reaping his reward, and that reward will be justly shared by those who aid him in his honorable course.

